# Phase controlled SERS enhancement

**DOI:** 10.1038/s41598-018-36491-0

**Published:** 2019-01-24

**Authors:** Yuanhui Zheng, Lorenzo Rosa, Thibaut Thai, Soon Hock Ng, Saulius Juodkazis, Udo Bach

**Affiliations:** 1 State Key Laboratory of Photocatalysis on Energy and Environment, College of Chemistry, Fuzhou University, Fuzhou, Fujian 350116 China; 2 Materials Science and Engineering, Commonwealth Scientific and Industrial Research Organization, Clayton South, Victoria 3169 Australia; 3 The Melbourne Centre for Nanofabrication, 151 Wellington Road, Clayton, Victoria 3168 Australia; 4 Swinburne University of Technology, Centre for Micro-Photonics (H74), P.O. Box 218, Hawthorn, Victoria 3122 Australia; 5 Department of Engineering “Enzo Ferrari”, University of Modena and Reggio Emilia, via Vivarelli 10, I-41125 Modena, Italy; 6 Department of Materials Engineering, Monash University, Wellington Road, Clayton, Victoria 3800 Australia

## Abstract

Surface-enhanced Raman spectroscopy (SERS) has attracted increasing interest for chemical and biochemical sensing. Several studies have shown that SERS intensities are significantly increased when an optical interference substrate composed of a dielectric spacer and a reflector is used as a supporting substrate. However, the origin of this additional enhancement has not been systematically studied. In this paper, high sensitivity SERS substrates composed of self-assembled core-satellite nanostructures and silica-coated silicon interference layers have been developed. Their SERS enhancement is shown to be a function of the thickness of silica spacer on a more reflective silicon substrate. Finite difference time domain modeling is presented to show that the SERS enhancement is due to a spacer contribution via a sign change of the reflection coefficients at the interfaces. The magnitude of the local-field enhancement is defined by the interference of light reflected from the silica-air and silica-silicon interfaces, which constructively added at the hot spots providing a possibility to maximize intensity in the nanogaps between the self-assembled nanoparticles by changing the thickness of silica layer. The core-satellite assemblies on a 135 nm silica-coated silicon substrate exhibit a SERS activity of approximately 13 times higher than the glass substrate.

## Introduction

Surface-enhanced Raman spectroscopy (SERS) is a sensitive analytical tool that is capable of both detecting and identifying chemical and biological compounds at low concentrations^1–4^. This involves the use of plasmonic antennas - metal-dielectric composite nanostructures - to amplify Raman signals through a process known as surface-enhanced Raman scattering. A unique feature of these antennas is their ability to support localized surface plasmon resonances, light-driven coherent oscillations of free electrons in the structures. The excitation of surface plasmons results in a strong electromagnetic (EM) field enhancement in the close vicinity of the metal surfaces. The largest localized field enhancement usually occurs near sharp asperities or corners associated with metal films or tips^5–8^ and within nanoscale gaps between metal nanoparticles (NPs)^1–4,8^, typically referred to as “hot spots”. The SERS intensity scales with the product of the localized EM field intensities at the excitation and Raman scattering wavelengths^9^. The SERS enhancement factor at a given location within a plasmonic hotspot is given by the product of the electric field (|E^2^|) enhancement at the excitation wavelength and the electric field (|E^2^|) enhancement at the scattering wavelength (usually Stokes but can be the anti-Stokes)^10^:

1 $$E{F}_{SERS}=\frac{{|{E}_{loc}({\lambda }_{ex})|}^{2}{|{E}_{loc}({\lambda }_{R})|}^{2}}{|{E}_{0}({\lambda }_{ex}){|}^{4}}$$

where E_0_ and E_loc_ are the incident field and localized field strengths at the hot spot respectively, and λ_ex_ and λ_R_ are the excitation and Raman scattering wavelengths. SERS enhancement factors are typically observed on an order of 10^4^–10^6^ and were reported to become as high as 10^8^–10^14^ in special cases^11^. Much work has been undertaken into the tailoring of plasmonic antennas’ composition^5^, dimension^12^, spacing/positioning^13^, configuration^12^, interparticle gap^1,2,12,14^ and porous features^5,6,15^ within the structure to achieve the maximum near-field enhancement^8^.

Beyond the tailoring of plasmonic antennas themselves, there is a search for the change of their external environments that can further increase the amount of the field enhancement. One strategy is to excite the plasmonic antennas through a dielectric material rather than directly through air when they are deposited on a transparent dielectric substrate^16^. The excitation through the dielectric material (a “back-side” SERS) generates a larger SERS signal. This is due to the enhancement of the electric field accounted for by the transmission and reflection coefficients and their sign change which are dependent on the propagation direction of light: from low-to-high refractive index or vice versa^16^. Another strategy is to integrate metallic and dielectric NPs into morphologically defined hybrid arrays^10,17^. Hong *et al*. showed that the localized EM field strength provided by metal NP assemblies could be further enhanced by the delocalized array modes of dielectric NPs^9,17^. The third strategy takes advantage of optical interference layers that allow for the engineering of antennas’ radiation characteristics to achieve maximum field enhancement^18^. The interference effect for SERS enhancement was first reported by Blue *et al*. for explaining the SERS performance of pyrazine thin layer on a silver film as a function of pyrazine thickness^19^. The concept of using optical interference layers for SERS sensing was recently introduced by Shoute *et al*.^20^. In their study, they reported the use of an interference substrate composed of a silver island film, a silica dielectric spacer and a silicon reflector as a SERS substrate. It has been shown that the SERS intensity profile of the molecules adsorbed on the silver island film follows the pattern of the interference peak. A similar phenomenon was also observed by the group of Gordon using a system consisted of silver nanoprisms on a gold ground plane (reflective layer) spaced by a TiO_2_ dielectric layer^21^. Two different SERS enhancement mechanisms based on the optical interference mechanism^20^ and the near-field enhancement mechanism^21^ were respectively proposed by the groups to interpret the relationship between SERS intensity and the thickness of dielectric spacer.

In this work, a concept of utilizing a synergistic effect of “back side” effect together with an optical interference for SERS enhancement was developed. This is achieved by combining strong near-field-coupled core-satellite assemblies with an optical interference substrate (i.e., a silica layer on the top of a silicon substrate) that allows the modulation of the local field enhancement at the hotspot. In this geometry, the hot spots have contribution of the “back side” excitation occurring for the reflected light at the inner silicon-silica interface^16^. We studied the SERS activity of the core-satellite nanostructures on the optical interference substrates using benzenethiol as a model analyte and found that the Raman signals of benzenethiol is modulated with the silica thickness and amplified by more than one order of magnitude by tuning the silica thickness to the optimum value compared with glass supporting substrates. A simulation model that takes into account the interference, the reflection and transmission coefficients, and phase changes was used to investigate the local field enhancement. In the simulation, we accounted for realistic SERS excitation and detection with an objective lens with numerical aperture (NA) of 0.75. It is found that the modulation in the SERS intensity is due to the field enhancements at the hot spots within the assemblies originated from (1) constructive interference between the light reflected from silica-air and silica-silicon interfaces; (2) creation of the hot spot at the nanogap of differently sized NPs within the assemblies. The effect of the local EM field enhancement on the nanogaps on the exit surface (“back-side”) is realized^16^, in this case, for the reflected light from the silica-silicon interface and is controlled by silica spacer thickness. This makes such geometry better suited for standard retro-reflection SERS measurements. SERS emitted light into a large solid angle from the near field patterns was collected using a high-NA optical detection system and the collection efficiency could be further increased following the relation between solid angle and NA, which is close to linear^22^.

## Results

### Mechanism of phase controlled SERS enhancement and SEM analysis

**Figure 1:**
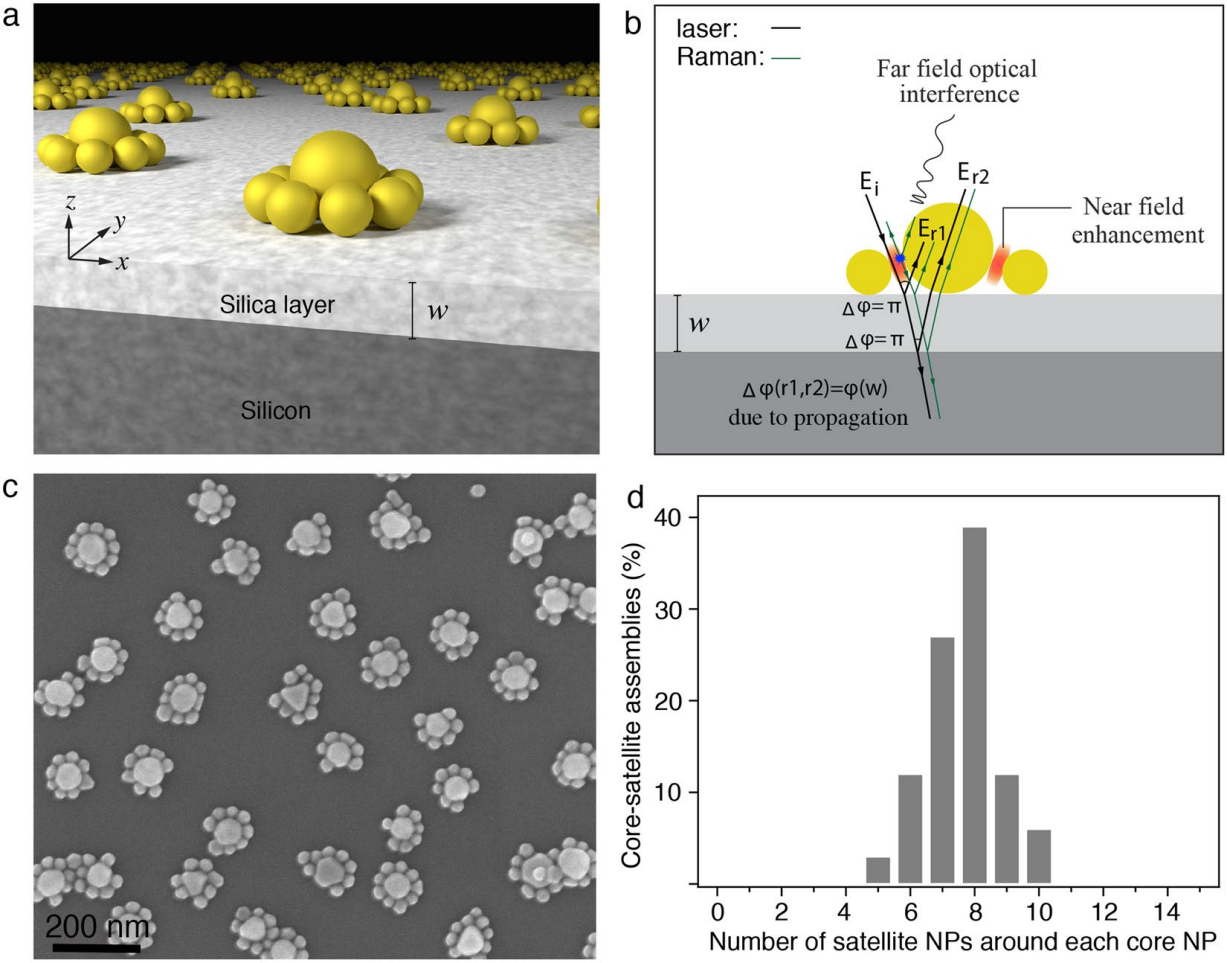
Core-satellite nanoassemblies on an optical interference substrate and their mechanism for SERS enhancement. (**a**) Schematic representation of 60 nm-30 nm core-satellite AuNP assemblies on an optical interference substrate composed of a silica spacer (silica thickness: *w*) and silicon reflector and (**b**) the SERS enhancement mechanism through phase control of the light. When the laser beam hits the substrate, reflection occurs at the air-silica and the silica-silicon interfaces. The reflected light from the silica-silicon interface and the incident wave create a phase-matched condition for the interference at the location of hot spots (near field) and excite the molecular vibrations. This results in emitting Raman photons coherent with excitation, which also undergo the near field enhancement at hot spots. E_i_: incident wave and E_r1_ and E_r2_: reflected waves from the top and bottom interfaces of the silica spacer. Note, this is a schematic representation of the optical interference of the reflections from air-silica and silica-silicon interfaces mediated by the NPs. (**c**) Scanning electron microscopy (SEM) image of 60 nm-30 nm core-satellite AuNP assemblies fabricated on a 110 nm silica-coated silicon substrate through a two-step self-assembly method that involves the electrostatic self-assembly of DNA-modified core NPs onto the positively charged silica surface and DNA-directed self-assembly of the complementary DNA modified satellite NPs onto the cores, and (**d**) statistical analysis of the number of satellite NPs surrounding each core NP.

Figure 1a schematically shows the configuration of the SERS sensor - an array of randomly distributed core-satellite AuNP assemblies where a larger core (average diameter: 60 nm) is encircled by smaller satellite NPs (average diameter: 30 nm) on an optical interference substrate that consists of a silica dielectric spacer with thickness of *w* on a silicon wafer. The optical interference occurs due to silica dielectric spacer on the silicon wafer (Fig. 1b). As shown in Fig. 1b, when the laser beam is incident on the substrate there are two reflection processes at the air-silica and the silica-silicon interfaces. At each interface, the incoming wave splits into two components (i.e. transmitted/reflected). The reflected light beams from the air-silica and silica-silicon interface create a phase-matched condition at the hot spots depending on the thickness, *w*. More specifically, both of the reflections undergo a π-phase change at the interfaces due to reflectance into low (air and silica) refractive index medium from that of higher index (silica and Si), respectively. The reflection from the silica-silicon interface propagates an extra distance in the dielectric medium to reach the hot spot between NPs on the surface. This causes an additional phase shift, which is dependent on the thickness of the dielectric spacer, *w*. By controlling the thickness of the dielectric layer, a constructive interference between the incident light and the light reflected from the silica-silicon interface can be achieved. Reflections and near field (nanogaps) contributions excite the Raman-active modes of molecular vibrations at the hot spots. Since the Raman scattering occurs simultaneously with excitation, they also undergo the same phase shift and enhancement as described above (shown schematically in Fig. 1b). Contribution of the propagation phase in the spacer (a far-field effect) will only have the π-phase shift upon reflection from a larger refractive index inner surface (Si in this study). This can be exploited to decrease the required thickness of the spacer.

The core-satellite nanostructures illustrated in Fig. 1a were fabricated through a two-step self-assembly method^4^. The DNA-modified core AuNPs (average diameter: 60 nm) were electrostatically adsorbed onto a 3-aminopropyltriethoxysilane (APTES) modified optical inference substrate, followed by the self-assembly of the complementary DNA-functionalized satellite NPs (average diameter: 30 nm) onto the pre-assembled cores via DNA hybridization. The successful self-assembly of the core-satellite nanostructures requires the neutralization of surface charges on the optical interference substrate prior to the assembly of the satellite NPs in order to avoid their non-specific adsorption onto the substrate. This was achieved by reacting the surface confined amino-groups with 4, 7, 10, 13-tetraoxatetradecanoic acid N-succinimidyl (mPEG_4_-NHS) ester, forming a PEG antifouling layer^23^. This two-step self-assembly method allows us to achieve high levels of core-satellite nanostructures uniformly distributed on the optical interference substrate, producing a narrow distribution of SERS signals over the whole substrate^4^. Figure 1c shows a typical scanning electron microscopy (SEM) image of the produced core-satellite AuNP assemblies on a 110 nm silica-coated silicon substrate. An average of 8 satellite NPs assembled on each of the core particle (Fig. 1d) and the density of the core-satellite assemblies is approximately 25 particles/μm^2^.

### Surface plasmon resonances and SERS activity

**Figure 2:**
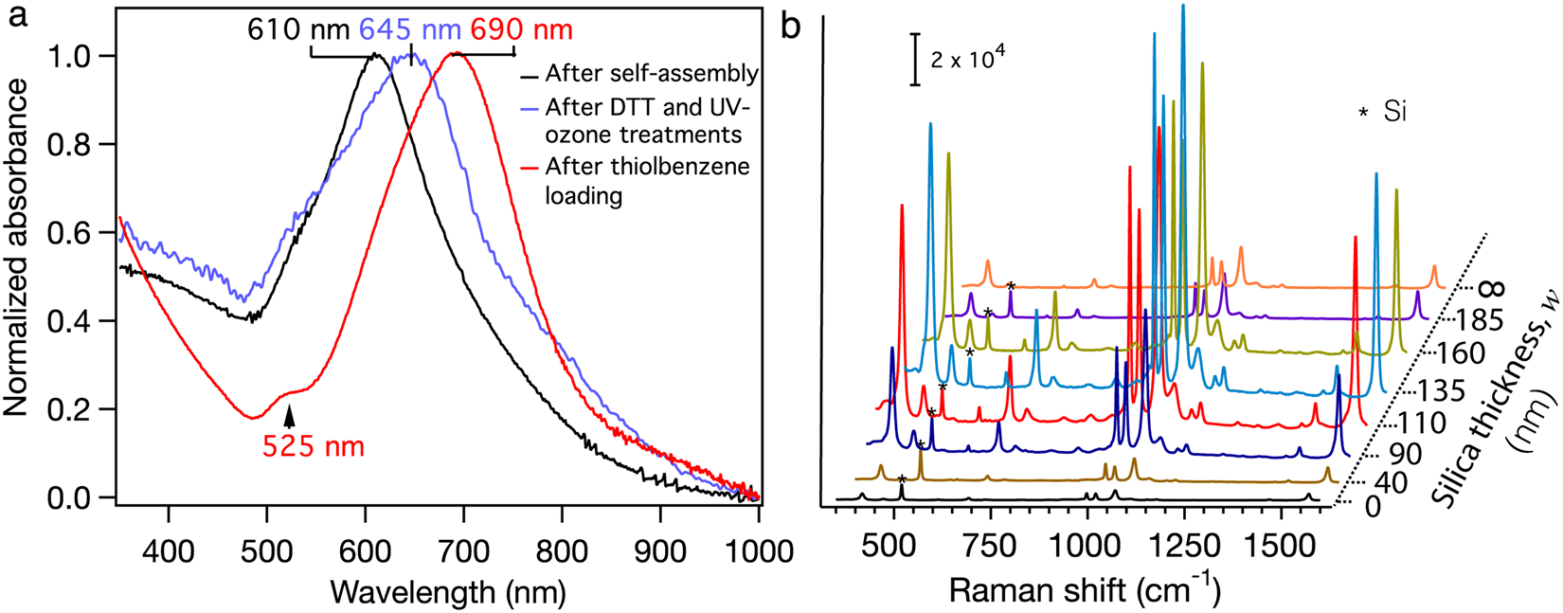
Optical property and SERS activity of the core-satellite nanoassemblies. (**a**) UV-vis absorption spectra of the core-satellite nanostructures on a glass substrate after self-assembly, dithiothreitol (DTT) and UV-ozone treatments, and benzenethiol loading (loading concentration: 1 μM; time: 12 h), and (**b**) silica thickness dependent SERS spectra of benzenethiol adsorbed on the core-satellite AuNP assemblies.

Figure 2a shows the optical properties of the assemblies on glass slides following the same self-assembly procedure. The self-assembled two-dimensional (2D) core-satellite nanostructures exhibit a broad surface plasmon resonance band with maximum absorbance at 610 nm and a shoulder at around 525 nm. The main band is ascribed to the in-plane (*xy* plane) plasmonic modes of the 2D core-satellite nanostructures, while the shoulder the out-of-plane (*xz* or *yz* plane) plasmonic mode^24^. For the subsequent SERS experiments, the surface-confined DNA strands were displaced with dithiothreitol (DTT) and then removed by UV-ozone treatment to achieve pristine core-satellite nanostructures. The pristine core-satellite AuNPs show a main surface plasmon resonance band at 645 nm and a shoulder at 525 nm. The redshift of the main plasmon resonance peak is ascribed to the decrease of interparticle gap, as DTT molecules are much shorter than the DNA strands. Upon the benzenethiol loading, the surface plasmon band splits into two distinguished peaks centred at 525 nm and 690 nm. The absorption peaks of benzenethiol are at 236 nm and 270 nm (see Supplementary Fig. S1). Since the absorption peaks of benzenethiol are non-resonant with the surface plasmon absorption peak at 645 nm, it should not contribute any redshift of the surface plasmon resonance. The redshift of the surface plasmon resonance from 645 nm to 690 nm is believed to be due to the increase refractive index arising from the benzenethiol adsorption (Note: The refractive indices of air and benzenethiol are 1 and 1.588 at 20 °C, respectively)^4^. It has been shown that the in-plane modes are more sensitive to the change in interparticle gaps and refractive index surrounding the core-satellite nanostructures^25,26^. This explains the significant red-shift of the in-plane modes rather than the out-of-plane mode upon the DTT and UV-ozone treatment and benzenethiol loading. Figure 2b shows the silica thickness dependent SERS spectra of benzenethiol adsorbed on the core-satellite assemblies excited by a 782 nm laser. The characteristic Raman peaks at 417, 468, 693, 996, 1022, 1071, and 1570 cm^−1^ of benzenethiol were observed^4^. The SERS intensity of these peaks reached a maximum with the increase of silica thickness at 135 nm and then decreased at a higher silica thickness.

To precisely describe the physical picture shown in Fig. 1b, we developed a model that takes into account the interference and phase change of the E-field upon reflection for the simulation of the near field enhancement at hot spots and the Raman scattering power fraction collected by the objective with NA = 0.75. The magnitude of the local-field enhancements is defined by the constructive interference (phase matching) of the incident and reflected light from the air-silica and silica-silicon interfaces at the hot spots. Accordingly, the SERS intensity (*I*_*SERS*_) scattered by the nanostructure is expressed as

2 $${I}_{SERS}({\lambda }_{SERS})=\alpha \eta ({\lambda }_{R},w)I({\lambda }_{ex}){\int }_{0}^{V}[|E({\lambda }_{ex},w){|}^{2}|E({\lambda }_{R},w){|}^{2}/|{E}_{0}{|}^{4}]dV$$

where *E*_0_ and *E(λ*_*ex*_ *or λ*_*R*_) are the incident field and local E-fields at excitation and Raman scattering wavelengths, respectively. *η(λ*_*R*_, *w*) is the Raman scattering power fraction for silica thickness of *w* collected by the objective and *I(λ*_*ex*_*)* is the excitation wave intensity. Constant *α* is a fitting parameter to match the measured and calculated SERS power and accounts for the number of contributing scatters at the hot spots. *V* is the collective volume of all the hot spots, evaluated by including the integral of all the volume elements whose intensity exceeds 1000 times the source intensity. This threshold intensity is chosen, as the field enhancement intensity lower than this value has almost no contribution to SERS intensity^27^. This is in good agreement with evaluation of SERS intensity contributions from hot spots^28^. The two main factors of the equation, namely *η(λ*_*R*_, *w)* and ${\int }_{0}^{V}[|E({\lambda }_{ex},w){|}^{2}|E({\lambda }_{R},w){|}^{2}/|{E}_{0}{|}^{4}]dV$, reflect the two fundamental effects included in the model, that is the far-field optical interference and the near-field enhancement, respectively. Uniqueness of this type geometry is in a thin spacer required due to π-phase shift of refection at silica-Si interface and that a hot spot is created at the “back-side” for the reflected beam^16^. The additional “back-side” SERS enhancement is due to the propagation direction dependent transmission and reflection coefficients and their sign change at the silica-Si interface. The intensity |E|^2^ shown in the widely used generic expression (Eqn. 2) does not reveal the phase importance of the interfering E-fields.

### 3D-FDTD Simulations

**Figure 3:**
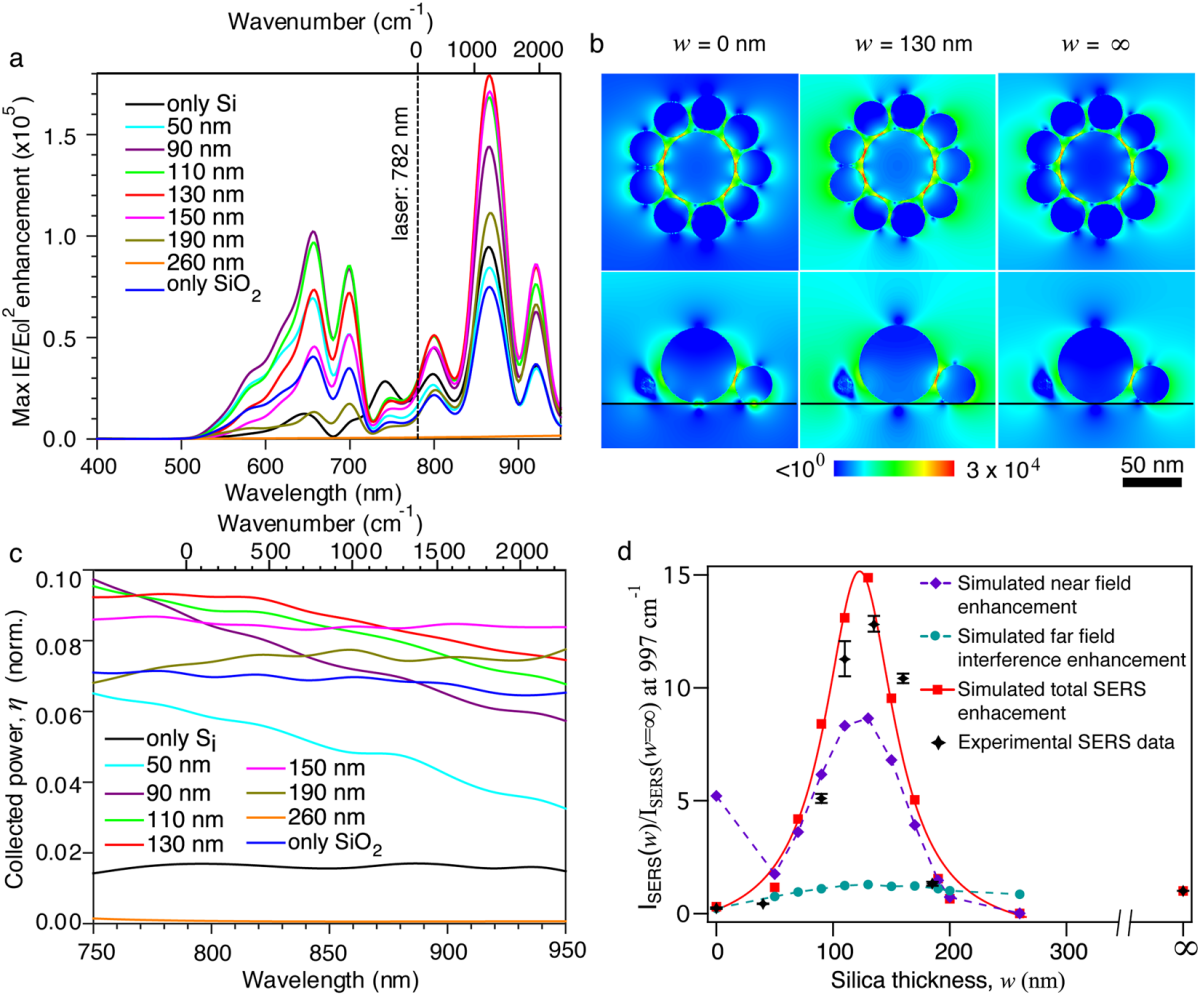
Simulation results. (**a**) Maximum electric field intensity (|E/E_0_|^2^) enhancement at mid-gap between core and satellites as a function of SiO_2_ thickness *w*. (**b**) Spatial distribution of electric field intensity enhancement at 782 nm wavelength for only Si (*w* = 0, first column), *w* = 130 nm (second column), and only glass (third column). The substrate-air interface is depicted by a black line, and the figures depict (top) the *xy*-plane cut bisecting the nanogaps and (bottom) the *xz*-plane cut. (**c**) Collected far-field Raman scattering power fraction *η* (normalized, i.e., for one molecule) by the objective with NA = 0.75 and (**d**) theoretical (solid red line) and experimental (black star) SERS intensity at 997 cm^−1^ as a function of the silica thickness. The experimental SERS data for each sample is a mean value of measurements from five random spots. Note that the dash lines are simulated near field enhancement (purple) and far field interference enhancement (green) at 848 nm (997 cm^−1^) a function of the silica thickness (Eqn. 2).

Figure 3 shows the breakdown of the near- and far-field effects. The maximum E-field intensity enhancement in the 1 nm gaps between core and satellites has been estimated in Fig. 3a. The produced core-satellite nanostructure has six major plasmonic extinction peaks at around 650 nm, 700 nm, 750 nm, 800 nm, 868 nm and 920 nm. Further calculation of the induced charge shows that the core-satellites ensemble excitation gives rise to a ring-like arrangement of dipoles (i.e. circular oscillation of surface charges, see Fig. S2 in Supporting Information), referred to as magnetic modes^24,29^. It has been reported that magnetic modes are unable to directly couple to the incident radiation and hence are not observed in the absorption spectra in Fig. 2a (i.e., they are dark modes)^24^. The dark modes are of essential importance for the generation of highest local fields and for the spectroscopic performance of a plasmonic nanostructure^30^. The main resonance of the magnetic mode appears at 868 nm, where the charge distributions induced in the satellites are in opposite phase with the core. Minor maxima appear due to local charge distribution shifts giving rise to secondary resonances. The plasmonic resonance peaks at 650 nm and 700 nm match well 633 nm laser and the Raman peak at 997 cm^−1^ (700 nm), respectively, while those at 800 nm and 860 nm match well 782 nm laser and the Raman peak at 997 cm^−1^ (848 nm), respectively. This is in good agreement with the excitation wavelength dependent SERS results (see Fig. S3 in Supporting Information). In this work, we are interested in understanding the SERS enhancement at near infrared (i.e., 782 nm) excitation. As shown in Fig. 3a, there is an enhancement region for *w* between 0 and 260 nm with maximum at 130 nm when excited at 782 nm.

Interference from air-silica and silica-silicon layers with corresponding π-phase shifts and propagation length in silica spacer constructively interferes at the location of a hot spot. The reflection coefficients of interfaces define the light E-field amplitudes. It was recently demonstrated that an additional mechanism of enhancement exists when radiation passes from a higher to a lower refractive index medium due to the same coefficients^16^. For the experimental situation discussed here, the light is propagating from silica into air (onto a hot spot) an additional enhancement around 2 will occur at that location which is given as t^4^ where the field transmission coefficient^16^:

3 $${\rm{t}}=2{{\rm{n}}}_{{\rm{i}}}/({{\rm{n}}}_{{\rm{i}}}+{{\rm{n}}}_{{\rm{t}}})=3/2.5=1.2$$

is defined by the refractive indices of the media where transmitted and incident light propagates, n_i_ = 1.5 (silica) and n_t_ = 1 (air), respectively^16^. The coefficient t is for the E-field amplitude, and for the intensity of incident and scattered light the enhancement is t^4^ = 2. This shows that by using larger refractive index spacer further enhancements can be reached due to this mechanism, which is not harnessed in practical applications and can be easily realised using properly engineered substrates. It is noteworthy, that when refractive index of the spacer is higher, it is necessary that it would still be higher than that of the lower substrate (here, Si). In such case one can benefit from the π-phase shift for the reflected light (a thinner spacer is required). A spectrally broad performance of the enhancement was recently demonstrated by ellipsometry on different substrates^31^. This is important for estimation the augmented enhancement at the wavelengths of excitation and Raman scattering. A more elaborated account of light field interference in the presence of anisotropy in SERS was studied using effective medium theory to describe the NP covered surface^32^. The constructive interference due to the silica layer, controlled by the layer thickness, and the enhancement (Eqn. 3) couples with the plasmonic enhancement effect in the near field, amplifying it. This is shown clearly in Fig. 3a, as the strongest amplification is reached at the wavelengths closer to the main plasmonic resonance at 860 nm.

It is interesting to examine the field distribution in the vicinity of the nanogaps to estimate the character of the scattering emission. The evaluation is done at the laser excitation wavelength of 782 nm (Fig. 3b). The spatial electric field distribution (|E|^2^) of the core-satellite nanostructures at an excitation wavelength of 782 nm (Fig. 3b) shows that the maximum electric field enhancement and the volume of the hot spots vary when changing the supporting substrate. It can be noticed that the hot spots are located within the core-satellite interparticle gaps, with a pattern following the orientation of the gaps with respect to the incident light polarization (*x*-axis). Three cases are shown in Fig. 3b: *w* = 0 nm (silicon, first column) *w* = 130 nm (SiO_2_ on silicon, second column) and *w* = ∞ (glass (SiO_2_), third column). The case for *w* = 130 nm shows the highest electric field enhancement in the gaps, as predicted. It is remarkable that for all silica thicknesses, the hot-spot pattern remains very consistent. The only noticeable variation is in the maximum values. The final near-field enhancement as a function of silica thickness is obtained by integrating the EM field at the excitation and Raman wavelengths over the hot spots and plotted in Fig. 3d (dash purple line).

The scattered light is recorded via a set of electric field monitors arranged around the structure, integrating the field intensity separately and calculating its fractions emitted into air, glass, and silicon media. The air fraction integral is aperture filtered to simulate the acceptance cone of the NA = 0.75 objective used to measure SERS. Figure 3c shows the collecting efficiency of back scattered Raman photons by the NA = 0.75 aperture for different silica thickness. For the core-satellite nanostructure on silicon, less than 2% scattered power for the excitation and Raman (997 cm^−1^) photons is collected (of total 20% scattered in air), increasing up to 8–9% for *w* = 130 nm (of total 40%), and settling at 7% around 200 nm, beyond which the effect of the reflection from silicon goes out of phase. The collection efficiency plateaus between 110 and 150 nm thickness and is fairly consistent with wavelength. The Raman scattering power fraction collected by the objective as a function of silica thickness is plotted in Fig. 3d (dash blue line).

Based on the local field enhancements and the collection efficiency of the scattered Raman photons, we calculated the theoretical SERS scattering power at the wavelengths of 782 (laser excitation wavelength) and 848 nm (Raman peak at 997 cm^−1^) using eqn. 2. Figure 3d compares the theoretically predicted (solid red line) and experimentally measured (black stars) SERS intensity at 997 cm^−1^ as a function of the silica thickness. The experimental SERS data for each substrate is an average value of five measurements. All these samples show a standard deviation (σ) of less than 20%, which is much smaller than that of a lithographically produced Klarite SERS substrate (σ = 45%)^4^. This indicates a good homogeneity of the SERS intensity over the sample surface of the fabricated SERS substrates. Since the experimental conditions for the fabrication of the core-satellite assemblies and for the analyte loading are identical for all samples, we assume that each core-satellite nanostructure accommodates the same number of analyte molecules for all samples. We define the molecule number as N in our simulation. The value N is cited as the value of the arbitrary constant α that verifies the SERS equation (Eqn. 2), in the hypothesis that all the molecules are equally contributing. As shown in Fig. 3d, the experimental relationship between the SERS activity and the silica thickness is theoretically reproduced. With the increase of silica thickness, the SERS scattering power reaches a maximum before decreasing. The theoretical SERS scattering power maximizes at silica thickness of around 130 nm. The expected SERS signal ratio between the *w* = 135 nm and *w* = ∞ case is ~13. For complete destructive interference cases (*w* = 0 or 260 nm), the near field enhancements are largely suppressed. This results in two SERS intensity minima (Fig. 3d). Theoretically, the SERS intensity drops to almost zero when the silica thickness reaches a value that matches the complete destructive interference (i.e., *w* = 0 or 260 nm).

A fundamental feature of the simulation model developed here is the inclusion of the enhancement contribution both at the excitation and at the SERS wavelengths. The effect predicts that a doubling in the local field intensity occurs as light passes from glass to air (Eqn. 3). The core-satellite structure enables multiple resonances, as seen in Fig. 3a, as the phase relationship between the core and the satellites varies with wavelength, and is affected by the wavelength at which maximum intensity occurs at the hot spots. The NA aperture accounts for the efficiency of collection at the SERS wavelength. If a 3D specific NP arrangement radiates into a specific aperture cone, there is a possibility to use a matching objective to maximize the Raman signal collection efficiency; this would greatly improve the SERS signal-to-noise ratio, aiming for single-molecule detection.

## Discussion

We have demonstrated the phase control for SERS enhancement using a combinatory system of self-assembled AuNP core-satellite nanostructures and an optical interference substrate as a SERS substrate. The strong near-field coupled core-satellite nanostructures were fabricated through bottom-up self-assembly. Their SERS activity is modulated by the thickness of the interference layer. The core-satellite assemblies on a 135 nm silica-coated silicon substrate exhibit a SERS activity of approximately 13 folds higher than the glass supporting substrate. A simulation model that examines the local-field enhancement including the interference and phase change effects at the excitation and at the Raman wavelengths on SERS enhancement has been developed to understand the relationship between the SERS activity and the silica thickness. The simulation shows that the core-satellite nanostructures exhibit three near-field type surface plasmon resonances at 800 nm, 860 nm and 920 nm, arising from dipole resonant modes of the ensemble. The intensity of these peak enhancements is found to rely on the thickness of the interference layer. This is due to the local-field enhancements at hot spots within the assemblies at the silica-air interface. The interference in the silica spacer provides the phase control (hence a far-field propagation effect). A real collection power of the used detection system was used in modelling. By accounting for both near and far field effects, the experimental relationship between the SERS activity and the silica thickness is theoretically reproduced.

## Methods

### Synthesis of AuNP-DNA conjugates

60 nm AuNP-DNA conjugates were synthesized through a salt-aging method^33^. In a typical procedure, 1 mL of 60 nm citrate-stabilized AuNP solution (Ted Pella) with optical density of 1.0 was concentrated to 100 μL by centrifugation (3200 rpm, 40 min; Eppendorf 5415 R centrifuge). 5 μL of 2.0% polyoxyethylene (20) sorbitan monolaurate (Tween 20) (Sigma-Aldrich), 30 μL of 0.1 M phosphate buffer (pH = 7.0) and 10 μL 100 μM of DNA-a solution (sequence: 5′-[HS]-T_15_ -TTA TGA CCC TGA TTA-3′, Fidelity Systems Inc.) A 2 M NaCl was gradually added into AuNP solution until the final NaCl concentration reaches 0.5 M, followed by adding 5 μl of 100 mM bis(p-sulfonateophenyl)phenylphosphine dihydrate dipotassium (BSPP: Sigma-Aldrich). The mixture was incubated at room temperature overnight and then washed with ultrapure water three times. After the final washing step, the 60 nm AuNP-DNA-a conjugates were redispersed in 250 μL ultrapure water. 30 nm AuNPs were functionalized with monothiolated DNA according to a method described earlier^4,34,35^. In a typical procedure, 1 ml of 30 nm citrate-stabilized AuNP solution (Ted Pella) with an optical density of 1.0 was concentrated to 100 μl by centrifugation (4,000 rpm, 45 min; Eppendorf 5415 R centrifuge). 5 μl of 2.0% Tween 20 (Sigma-Aldrich), 30 μl of 0.1 M phosphate buffer (pH = 7.0), 50 μl of 2.0 M NaCl, 10 μL of 100 μM *DNA-a’* solution (sequence: 5′-[HS]-T_15_-TAA TCA GGG TCA TAA-3′) and 5 μl of 100 mM bis(p-sulfonateophenyl)phenylphosphine dihydrate dipotassium (BSPP: Sigma-Aldrich) was added to the AuNP solution. The mixture was incubated at room temperature overnight; the AuNP-DNA colloidal solution was washed three times with ultrapure water. After the final washing step, the 30 nm AuNP/DNA-*a* conjugates were redispersed in in 200 μl of a buffer ([AuNP] ≈ 1.65 nM) of 0.05% Tween 20, 0.5 M NaCl and 20 mM K_2_HPO_4_/KH_2_PO_4_ (pH = 7.0).

### Fabrication of silica-coated silicon substrates and their APTES modification

Silicon wafers were thermally oxidized by flowing a mixture oxygen-nitrogen gas over silicon wafers in an 1100 °C furnace for a period of time. The silica thickness is varied by controlling the reaction time and determined by optical profilometer. The wafers were cut into 4 × 6 mm^2^ pieces. Silica-coated silicon with different silica thickness were modified with a layer of 3-aminopropyltriethoxysilane (APTES) by immersion into a mixture of APTES, water and ethanol with volume ratio of 2: 3: 95 for 1 h, washed with ethanol three times, dried under a stream of nitrogen and baked at 110 °C for 10 min.

### Synthesis of core-satellite nanostructures

Core-satellite nanostructures were fabricated through a two-step self-assembly method described earlier^4^. In a typical procedure, 20 μl of the 60 nm AuNP-DNA-*a* solution was placed on an APTES modified silicon or glass surface and incubated in a humidity chamber for 2 h. The substrate was washed three times with ultrapure water to remove nonspecifically adsorbed AuNPs, and dried under a stream of nitrogen. The substrate was subsequently immersed into 200 μl of buffered 4,7,10,13-tetraoxatetradecanoic acid N-succinimidyl (mPEG_4_-NHS) ester (Thermo Scientific Pierce) solution containing 0.5 mM mPEG_4_-NHS and 10 mM phosphate buffer (pH = 7.5) at 45 °C for 2 h to neutralize the surface charges of the amino groups. Following the mPEG_4_-NHS treatment, the substrate was washed three times with ultrapure water and then placed in a centrifuge tube containing 200 μl of the 30 nm AuNP/DNA-*a’* solution. The tube was placed in a 65 °C water bath that was allowed to slowly reach room temperature and incubated for 12 h, upon which the substrate was washed with a buffered saline solution containing 0.5 M NaCl and 20 mM K_2_HPO_4_/KH_2_PO_4_ (pH = 7.0) and 0.1 M ammonium acetate solution three times, respectively, and then dried naturally in air. Subsequently, the substrate was exposed to a 0.1 M dithiothreitol (DTT) solution, washed with a buffered saline solution containing 0.5 M NaCl and 20 mM K_2_HPO_4_/KH_2_PO_4_ (pH = 7.0) and a 0.1 M ammonium acetate solution three times and then dried under a stream of nitrogen. Following the DTT treatment, the substrate was exposed to UV-ozone at an oxygen flow rate of 3 L/min for 3 × 20 min. After the UV-ozone treatment, the substrate was immersed in 2 mL of 1 μM benzenethiol in ethanol solution and left for 12 h, upon which the substrate was rinsed with ethanol and then dried under a stream of nitrogen.

### Characterization

Scanning electron microscope (SEM) images of the self-assembled core-satellite nanostructures were taken with a field emission JEOL 7001 F SEM. Since conductive substrates are required for the SEM measurements, silicon wafers were used as supporting substrates for the self-assembly of the core-satellite nanostructures. For Raman and optical measurements, transparent glass slides were used as supporting substrates for the self-assembly of the core-satellite nanostructures. Absorption spectra were recorded using an Agilent 8453 UV-Vis spectrometer. Raman spectra were recorded using a Renishaw RM 2000 Confocal micro-Raman System equipped with a near infrared diode laser at a wavelength of 782 nm (laser power: 1.15 mW and laser spot size: ~1 µm). All Raman spectra were collected by fine-focusing a 50× microscope objective and the data acquisition time was 10 s.

### Theoretical modeling

The core-satellites are simulated with the 3D finite-difference time-domain method (3D-FDTD), by the Lumerical software (FDTD Solutions, Inc.). A single core-satellite nanostructure was simulated (60 nm core and 30 nm satellite) on a glass layer of thickness *w* on top an infinite silicon substrate. Gap width was set at 1 nm as per previous experiments^27^, resulting in nine satellites fit around the core. E-field monitors record the scattered light into Si, glass, and air. The air fraction is filtered through a round aperture to reproduce the NA = 0.75 objective used in the SERS measurement system. Perfectly matched layers (PMLs) absorb stray radiation, the mesh size of 0.2 nm and excitation is by an x-polarized wave from the air side, with wavelength from 400 nm to 950 nm. The glass thickness is varied from *w* = 0 (only Si) to *w* = ∞ (only glass). The material refractive indices are fitted from literature^36^.

## Electronic supplementary material

Supplementary Information
