# Supplementary material for: Phase controlled SERS enhancement
Source: Sci Rep. 2019 Jan 24;9:744. doi: 10.1038/s41598-018-36491-0 (PMC6346009; doi:10.1038/s41598-018-36491-0)
Supplement: Supplementary file 1 — Supplementary Information [file 41598_2018_36491_MOESM1_ESM.docx]

Supplementary Information

**Phase controlled SERS enhancement**

Yuanhui Zheng,^1,2,3^ Lorenzo Rosa,^4,5^ Thibaut Thai,^3,6^ Soon Hock Ng,^3,6^ Saulius Juodkazis,^3,4^ and Udo Bach^2,3,6^

^1^State Key Laboratory of Photocatalysis on Energy and Environment, College of Chemistry, Fuzhou University, Fuzhou, Fujian, 350116, China. ^2^Materials Science and Engineering, Commonwealth Scientific and Industrial Research Organization, Clayton South, Victoria 3169, Australia. ^3^The Melbourne Centre for Nanofabrication, 151 Wellington Road, Clayton, Victoria 3168, Australia. ^4^Swinburne University of Technology, Centre for Micro-Photonics (H74), P.O.Box 218, Hawthorn, Victoria 3122, Australia. ^5^Department of Engineering "Enzo Ferrari", University of Modena and Reggio Emilia, via Vivarelli 10, I-41125 Modena, Italy. ^6^Department of Materials Engineering, Monash University, Wellington Road, Clayton, Victoria 3800, Australia.

Email: Yuanhui.Zheng@fzu.edu.cn; [sjuodkazis@swin.edu.au](mailto:sjuodkazis@swin.edu.au); udo.bach@monash.edu

**1. UV-vis absorption spectrum of benzenethiol**

**
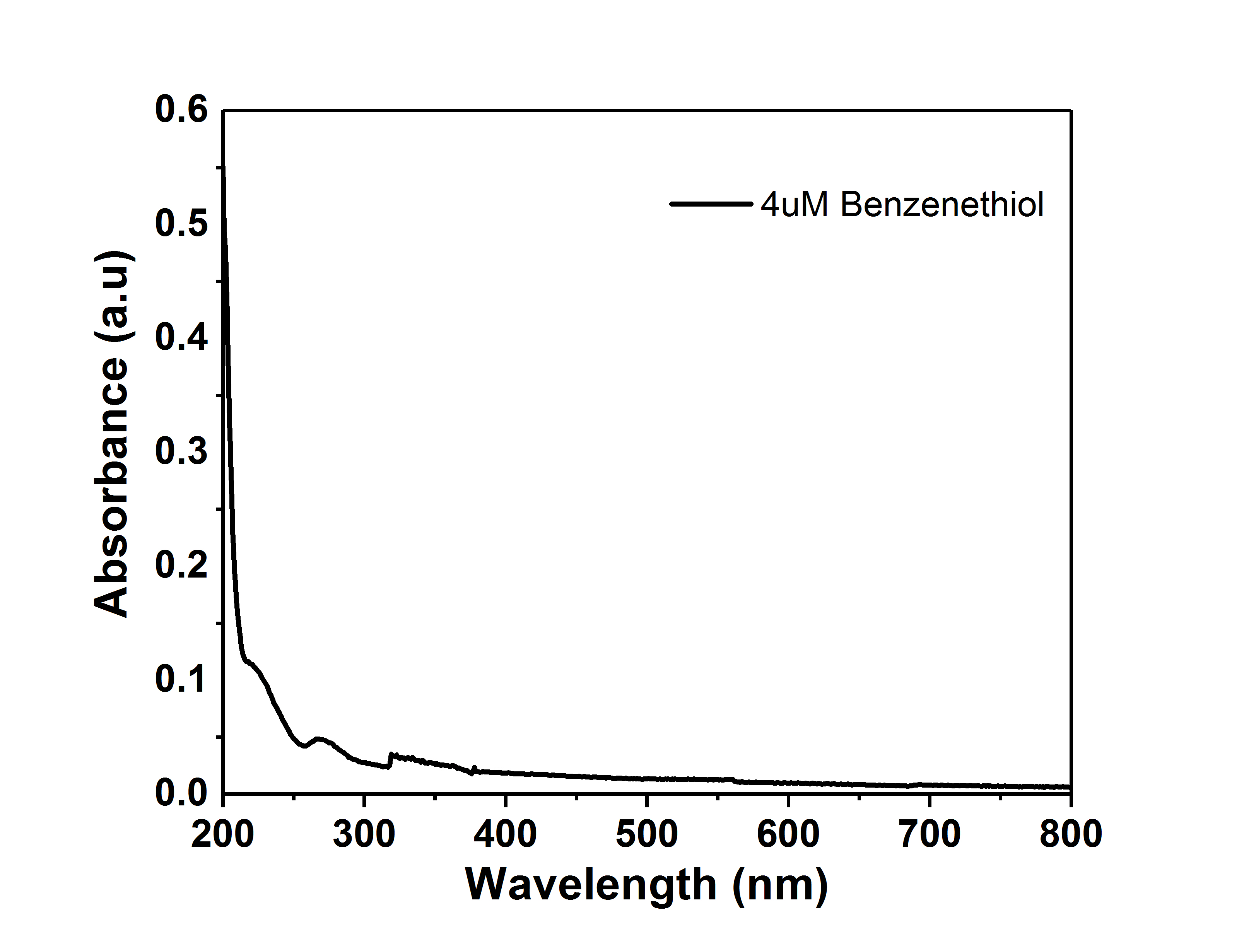
**

**Figure S1|** UV-vis absorption spectrum of 4 uM benzenethiol in ethanol. Note: the absorption peak of benzenethiol locates at ~ 260 nm. The jumps in the range of 300-400 nm are due to the change of lamps during the measurements.

**2. Understanding the theoretical plasmon resonances**

The 3D-FDTD simulation of the core-satellite nanostructure has been used to investigate the nature of the resonant modes of the ensemble by calculating the charge distribution on the gold nanospheres at the three plasmonic resonance peaks at around 800 nm, 860 nm and 920 nm.

The core-satellites ensemble is excited as a dipole mode, as shown in Fig. 1: the core charge follows the polarity of the exciting x-linear polarization and the satellites assume opposite local charge to balance. This results in a charge buildup around the gaps, which boosts the E-field enhancement.

The main resonance is at 868 nm, where the E-field distributions induced in the satellites are in straight opposite phase with the core.

Minor maxima appear due to local phase matching, as the satellites are not in straight phase opposition with the core, but the charge distribution shifts enough to give rise to a local buildup in a few of the gaps, resulting in secondary resonances.


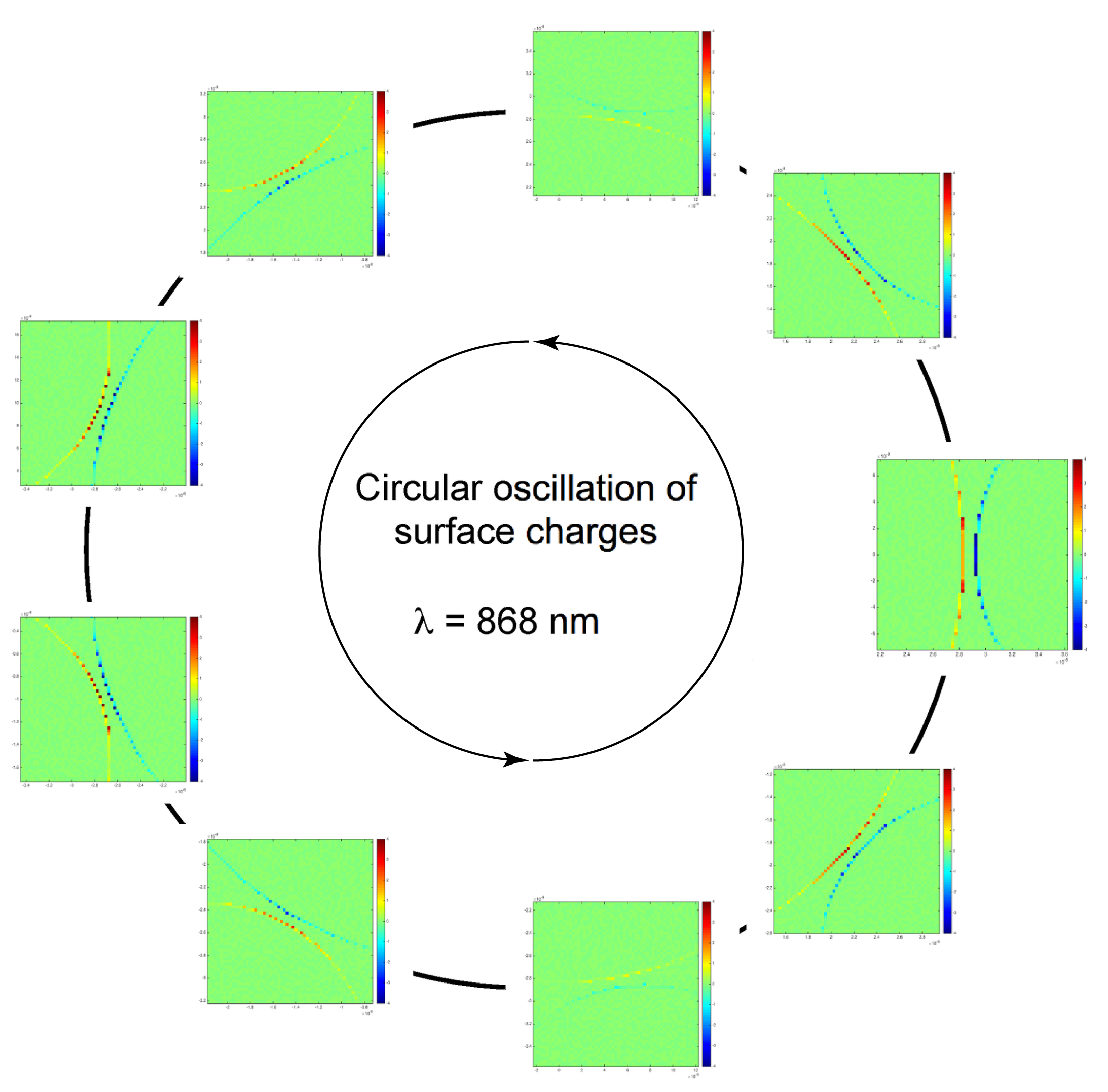


**Figure S2|** **The local gap plots depict the real part of the induced charge in proximity to the gaps between the core and the satellites.** The single plots show the circular oscillation of surface charges, with the resulting a magnetic mode.

**3. Excitation wavelength dependent SERS intensity**

**Figure S3|** **Excitation wavelength dependent SERS intensities of benzenethiol adsorbed on the core-satellite nanostructures supported by a 110 nm silica-coated silicon substrate**: 514 nm (8 mW; black), 633 nm (3.5 mW; purple) and 782 nm (1.15 mW; red). Note that all Raman spectra shown here were collected by fine-focusing a 50× microscope objective (laser spot size: ~1 µm) and the data acquisition time was 10 s.
